# Impact of Climate Change on the Composition and Bioactivity of *Inula viscosa* Essential Oils: In Vitro, *In Silico*, and ADME Analysis

**DOI:** 10.1002/fsn3.70845

**Published:** 2025-09-01

**Authors:** Abdelouahid Laftouhi, Amal Elrherabi, Ayoub Farihi, Fahd A. Nasr, Anouar Hmamou, Mohamed Adil Mahraz, Mohamed Bouhrim, Mohammed Al‐zharani, Ashraf Ahmed Qurtam, Noureddine Eloutassi, Abdslam Taleb, Mustapha Taleb

**Affiliations:** ^1^ Laboratory of Electrochemistry, Modeling and Environment Engineering (LIEME) Sidi Mohamed Ben Abdellah University, Faculty of Sciences Fes Morocco; ^2^ Bioresources, Biotechnology, Ethnopharmacology, and Health Laboratory, Department of Biology, Faculty of Sciences Mohamed First University Oujda Morocco; ^3^ Biology Department, College of Science Imam Mohammad Ibn Saud Islamic University (IMSIU) Riyadh Saudi Arabia; ^4^ Biological Engineering Laboratory, Faculty of Sciences and Techniques Sultan Moulay Slimane University Beni Mellal Morocco; ^5^ Environmental Process Engineering Laboratory, Faculty of Science and Technology Mohammedia Hassan II the University of Casablanca Casablanca Morocco

**Keywords:** antidiabetic activity, antioxidant activity, climate change, essential oils, molecular docking

## Abstract

Medicinal plants, rich in secondary metabolites, play a crucial role in drug development. Climate change, driven by industrialization, affects plant growth and the production of these metabolites. The research explores how climate change influences the phytochemical profile as well as the antioxidant and antidiabetic activities of *Inula viscosa.* The three samples were subjected to contrasting climatic conditions, ranging from a normal climate to progressively intensified combined heat and drought stress over 4 years (Sample 1 under normal seasonal temperature and rainfall; Sample 2 with a 5 C temperature increase and 50% reduced irrigation; and Sample 3 exposed to a 10 C temperature rise and 75% water deficit). Essential oils were extracted and evaluated for antioxidant activity (DPPH, ABTS, and β‐carotene bleaching) and their antidiabetic potential through the inhibition of α‐amylase and α‐glucosidase. In silico analyses, including molecular docking and ADME profiling, were performed, using AutoDockTools and SwissADME, to assess the potential of phytochemicals as inhibitors and their pharmacokinetic properties. The essential oils exhibited significant antioxidant activity, with Sample 2 showing the highest DPPH, ABTS, and β‐carotene bleaching activities. Additionally, notable antidiabetic effects were observed, with Sample 2 exhibiting the strongest inhibitory activity against α‐amylase and α‐glucosidase. Molecular docking studies revealed promising affinities of phytochemicals, such as caryophyllene oxide and α‐cuprenene, for α‐amylase and α‐glucosidase, supporting their potential as antidiabetic agents. These findings highlight the influence of climatic conditions on the biological activity of essential oils. *I. viscosa* essential oils from arid climates exhibit enhanced antioxidant and antidiabetic properties, with potential therapeutic applications. These effects are closely linked to climate‐driven changes in the chemical composition of the oils.

## Graphical abstract

This study explores the impact of climate change on the chemical composition and bioactivity of *Inula viscosa* essential oils. Samples cultivated under different climatic conditions in Taounate, Morocco, were analyzed for antioxidant and antidiabetic activities, supported by molecular docking and ADME predictions. Results revealed that oils from arid regions showed enhanced bioactivity, suggesting a strong link between climate variation and therapeutic potential.

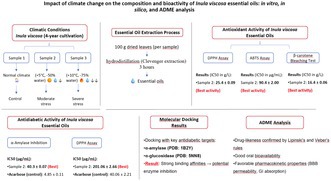

## 1 Introduction

Since the dawn of human civilization, various plant groups have been esteemed for their nutritional benefits, healing properties, and ability to sustain daily life (Bédoui et al. 2024; Rahmouni et al. 2024). Notably, medicinal plants hold particular significance due to their secondary metabolites and pharmaceutical attributes, making them indispensable in the pharmaceutical, medical, cosmetic, and nutritional industries (Hassan et al. 2012). According to the World Health Organization (WHO), around 80% of the global population depends on traditional medicine, particularly treatments based on medicinal plants. Ayurveda, a traditional system of medicine, verifies the use of approximately 45,000 plants across more than 21,000 species for medicinal purposes (Niazi and Monib 2024). Secondary metabolites contained in plants are widely employed in various fields, particularly in drug development. Their broad spectrum of bioactive compounds, limited toxicity, and affordability make these metabolites excellent candidates for driving innovation in drug discovery (Verpoorte and Memelink 2002). Many scientific investigations have shown that secondary metabolites play a key role in lowering the risk of several major health disorders such as diabetes, cancer, tuberculosis, ulcers, asthma, Alzheimer's disease, and cardiovascular conditions (Miller and Snyder 2012; Badraoui et al. 2023; da Costa et al. 2024; Jerada et al. 2024). Diabetes mellitus (DM) poses a major challenge to public health worldwide. The International Diabetes Federation (IDF) reported that in 2017, about 425 million adults aged between 20 and 79 years, representing 8.8% of the global adult population, were affected by diabetes. If current trends persist, projections suggest that by 2045, the number of individuals with diabetes will rise to 629 million adults aged 20–79 years, corresponding to approximately 693 million individuals aged between 18 and 99 years (Kumar et al. 2024). Similarly, an imbalance in the production of reactive oxygen species within the body can lead to elevated oxidative stress, which causes oxidative harm to cells and tissues and can aggravate chronic diseases such as diabetes, cancer, and cardiovascular disease (Tavares and Seca 2019). Climate change exerts a broad spectrum of negative impacts in multiple fields, particularly human health, water resources, air quality, soil integrity, microbial ecosystems, and plant life, along with their pharmaceutical components and secondary metabolites, affecting food security. Key contributors to climate change include the increasing world population, rapid industrial growth, and widespread application of chemical fertilizers and pesticides in farming. Climate changes encompass increasing temperatures, cold spells, droughts, and altered rainfall patterns. These shifts disrupt the normal functioning of humans, plants, and microbial communities, among others (Gupta et al. 2019). Today, the global population is experiencing an unprecedented demographic explosion, leading to intensified exploitation of natural resources (Adjonou et al. 2009) and exacerbating the impacts of the Industrial Revolution (Ubertosi et al. 2022). However, this surge in industrial activity poses significant threats to the environment, giving rise to various ecological problems (Akula and Ravishankar 2011), with climate change (Abaza et al. 2023) emerging as the most pressing concern. Climate change is currently a looming crisis, with the potential to trigger the extinction (Ackah Stéphane et al. 2019) of numerous species inhabiting our planet (Ahammed et al. 2023). Its severity is continuously escalating (Al‐Huqail et al. 2020), fueled by the relentless emission of greenhouse gases into the atmosphere, primarily due to industrial activities (Alaoui 2017). Experts warn that without intervention, the current trajectory of greenhouse gas emissions (Asghari et al. 2023) could result in a temperature increase of 5.3°C by 2100 (Aru et al. 2022). The consequences of climate change (Aslani et al. 2023) are further exacerbated by profound alterations in our planet's climate (Yang et al. 2024), leading to detrimental impacts on human health and the environment (Attou et al. 2022). Consequently, addressing climate change has become a paramount concern (Ayedegue et al. 2020) within the global scientific community (Baali et al. 2019), prompting urgent action at international, national, and local levels (Berardi and Jafarpur 2020). Finding effective and ambitious solutions (Bnouham et al. 2002) to mitigate the catastrophic consequences of climate change is imperative (Bouremani et al. 2023). Recent temperature increases of up to 5°C have been observed, affecting various plant species and their yields. These temperature fluctuations affect both plant secondary metabolites and growth by altering the metabolic pathways that control signaling, physiological functions, and defense responses Furthermore, climate change also impacts primary metabolites like amino acids, sugars, and intermediates of the Krebs cycle (Laccourreye and Maisonneuve 2019). In our previous study on *Inula viscosa* leaves, we found that climatic factors, such as rising temperatures and declining precipitation, significantly affected both primary and secondary metabolites. Key primary metabolites, including proteins and amino acids like phenylalanine and leucine, were reduced under climatic stress, with the highest levels observed in Sample 1 and the lowest in Sample 3. Ethanol‐extracted secondary metabolites showed varying trends, and essential oil yields increased in Sample 2 but declined in Sample 3 under stress. GC–MS analysis revealed distinct shifts in essential oil composition, with compounds like (E)‐nerolidol, α‐calcarine, cedr‐8(15)‐en‐9‐α‐ol, and guaiol being prominent. Mineral content generally declined, except for calcium and phosphorus. These results underscore the considerable effect of climatic conditions on the chemical profiles and adaptability of *I. viscosa* (Laftouhi, Slimani, et al. 2024). This study primarily aims to understand the physiological behavior of *I. viscosa* concerning climate change in terms of chemical compounds, antioxidant, and antidiabetic activity.

## 2 Materials and Methods

### 2.1 Study Area

**FIGURE 1:**
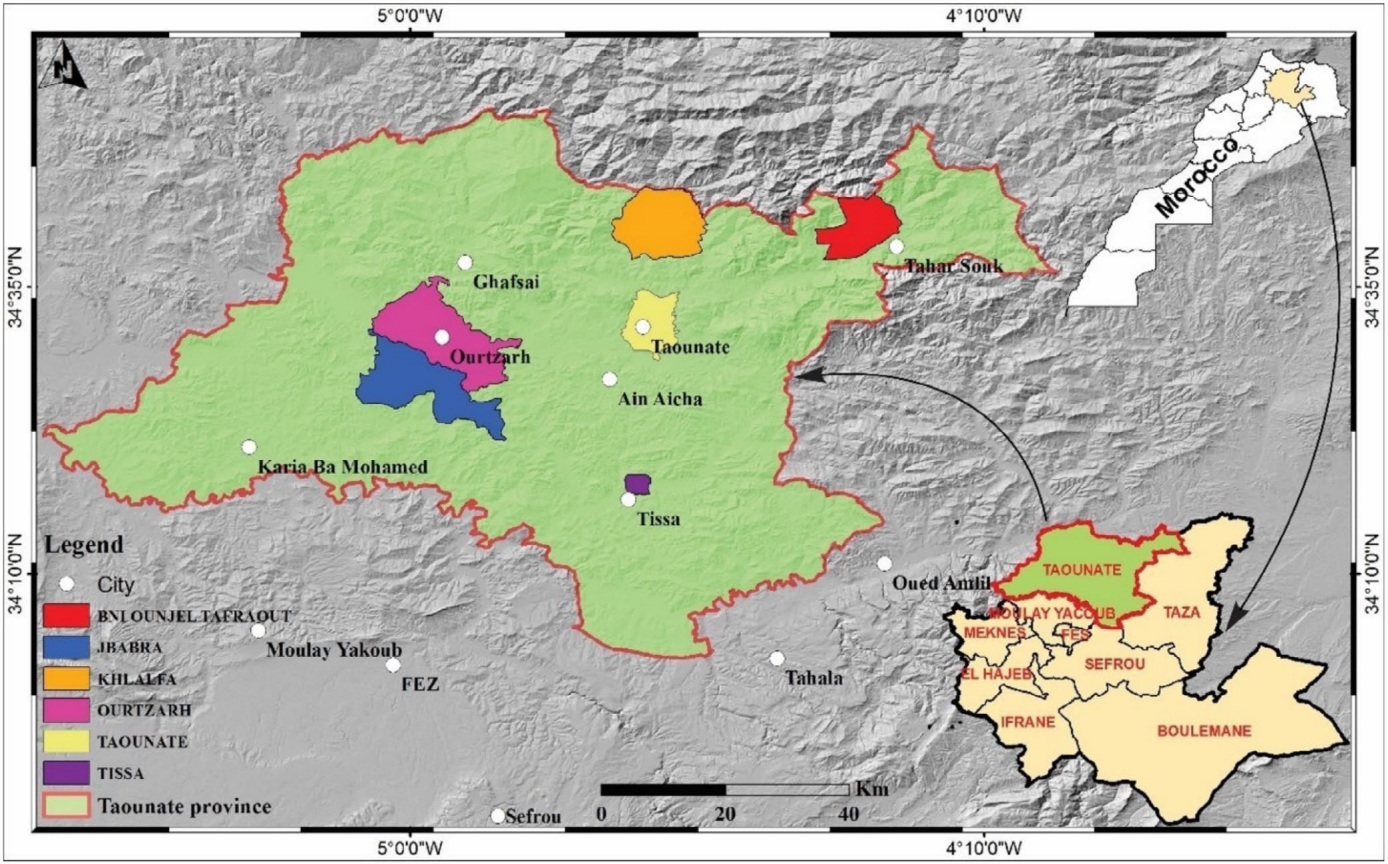
The study area.

Taounate is situated in northern Morocco, at the pre‐Rif level; it has a rural character (Figure 1). The total area of this region is approximately 5600 km^2^, which is divided into two distinct parts: the mountainous northern part where forests are located, and the southern part where agriculture is practiced. The altitude of this region varies between 80 m in the Ouerga bed and 1000 m in the mountainous northern part. It has a Mediterranean climate with an alternation of two seasons, one of which is humid and cold and the other dry and hot. The precipitation falls on average 790 mm with maxima, which can be around 2000 mm at Jbel Oudka. The difference in precipitation between the driest month and the wettest month is of the order of 100 mm. The average temperature is approximately 16.9°C. July is the warmest month, with an average of 26.5°C, while January is the coldest, averaging 9.1°C. It is important to note that during the summer season, temperatures can exceed 45°C and can drop to 2°C during the winter period (Hafsé et al. 2015; Laftouhi, Eloutassi, Ech‐Chihbi, et al. 2023; Laftouhi, Slimani, et al. 2024).

### 2.2 Climatic Planting Conditions

The plants used in this work originate from the Taounate region, which is located in the following coordinates: 34°32′9″ N 4°38′24″ W/34.53583° N 4.64000° W/34.53583; −4.64000. We calculated the 10‐year climatic balance of the Taounate region for temperature and precipitation, and then we cultivated three samples of *I. viscosa* from this region in different climatic conditions in a closed chamber for 4 years (Laftouhi, Eloutassi, Drioua, et al. 2023). Under defined experimental conditions, Sample 1 was maintained at typical seasonal temperature and rainfall levels. Sample 2 underwent an increase of 5°C in temperature, accompanied by a 50% reduction in irrigation, whereas Sample 3 was exposed to a 10°C temperature elevation and a 75% water deficit for four consecutive years. This specimen is cataloged as Voucher H20 (El‐Hilaly et al. 2003).

### 2.3 Extraction of Essential Oils

After the controlled cultivation of three distinct *I. viscosa* samples, the aerial parts of the plants (mainly the leaves) were harvested in the spring, specifically in April 2024. This period corresponds to the plant's optimal phenological stage, during which the concentration of secondary metabolites particularly volatile compounds is generally at its highest.

The harvested leaves were subjected to natural shade drying at room temperature for 10 days. This drying process, conducted away from direct sunlight, aims to minimize the degradation of thermosensitive and photosensitive compounds such as essential oils and flavonoids. This methodological choice is crucial for preserving the biochemical quality of the plant material prior to extraction.

To ensure the taxonomic validity of the studied species, a reference herbarium specimen (code H20) was identified by expert botanists at the National Scientific Institute of Morocco. This step is essential to avoid identification errors, especially given the intraspecific morphological variability of *I. viscosa* depending on local ecological conditions.

For essential oil extraction, an exact weight of 100 g of dried plant material was measured for each sample. The extraction was carried out by hydrodistillation using a Clevenger‐type apparatus for 3 h, following standard protocols in phytochemistry (Laftouhi, Eloutassi, Ech‐Chihbi, et al. 2023).

### 2.4 Antioxidant Activity of the Essential Oils

The radical scavenging activity was measured to evaluate the antioxidant potential of the samples using three distinct assays: the DPPH (2,2‐diphenyl picryl‐hydrazyl) test, the ABTS free radical scavenging test, and the carotene bleaching test, as described by Ainseba et al. and Pukalskas et al. All assays were performed in triplicate to ensure the accuracy and reliability of the results (Pukalskas et al. 2002; Ainseba et al. 2023). The DPPH test involves assessing the sample's ability to donate hydrogen atoms or electrons to the DPPH radical, causing the color to shift from purple to yellow, which is measured spectrophotometrically. The ABTS assay quantifies the reduction process of the ABTS radical cation to its non‐radical form by antioxidants, with the degree of decolorization indicating the scavenging ability. The carotene bleaching test evaluates the inhibition of the oxidation of carotene in the presence of linoleic acid, where the antioxidant activity is determined by the sample's ability to prevent the discoloration of carotene. These techniques offer a thorough assessment of the samples' antioxidant capabilities, leveraging different mechanisms of radical neutralization (Pukalskas et al. 2002; Ainseba et al. 2023).

### 2.5 Antidiabetic Activity of the Essential Oils

The inhibition tests involving the enzymatic activities of α‐amylase and α‐glucosidase are critical biochemical assays to assess the potential of a substance to inhibit these enzymes, which are essential for carbohydrate metabolism. The method for evaluating α‐amylase inhibition follows the protocol outlined by Elrherabi et al. (2023). The test mixture was prepared by combining 200 μL of phosphate buffer (0.02 M, pH 6.9), 200 μL of α‐amylase enzyme solution, and 200 μL of either the test extract or acarbose. This mixture was pre‐incubated at 37°C for 10 min. Subsequently, 200 μL of a 1% starch solution was added, followed by further incubation at 37°C for 15 min. The reaction was stopped by adding 600 μL of DNSA reagent, and the samples were then heated at 100°C for 8 min. After rapid cooling in an ice‐water bath, the mixture was diluted with 1 mL of distilled water. Absorbance readings were taken at 540 nm using a spectrophotometer. The percentage of enzyme inhibition was calculated according to the following formula:

$$\%\text{Inhibition}=\frac{\left(A_{\text{control}}-A_{\text{sample}}\right)}{A_{\text{control}}}\times 100$$

where, *A*_control_ is the absorbance of the reaction without the inhibitor and *A*_sample_ is the absorbance with the inhibitor. The IC_50_ value, indicating the concentration required to inhibit 50% of the enzyme activity, is determined graphically from the plot of inhibition percentage versus the logarithm of sample concentration. For α‐glucosidase inhibition, the procedure also follows Elrherabi et al. (2023). The reaction mixture includes 1 mL of 50 mM phosphate buffer, 0.1 mL of α‐glucosidase enzyme solution, and 200 μL of the extract or acarbose. After pre‐incubating at 37°C for 20 min, 0.1 mL of sucrose is added, and the reaction is terminated by heating at 100°C for 5 min. Then, 1 mL of GOD‐POD reagent is added, followed by a 10‐min incubation at 37°C. The absorbance is measured at 500 nm. The percentage inhibition is determined using the same formula as for α‐amylase. The IC_50_ is similarly obtained from the inhibition curve plotted against the logarithm of the sample concentration.

### 2.6 Molecular Docking Study

#### 2.6.1 Protein Preparation

Protein crystal structures for human α‐amylase (PDB: 1B2Y), human α‐glucosidase (PDB: 5NN8), and glutathione reductase (GR) (PDB: 3DK9) were sourced from the Protein Data Bank (PDB) (available online at www.rcsb.org). Each crystal structure was processed by removing water molecules and adding polar hydrogens along with Kollman charges using AutoDockTools (ADT; version 1.5.7). A grid box measuring 40 × 40 × 40 points with a spacing of 0.375 Å was generated to encompass both the active sites and surrounding regions of the proteins, centered at specified *x*, *y*, and *z* coordinates. The finalized macromolecules were saved in PDB format for subsequent molecular docking studies (Farihi et al. 2023).

#### 2.6.2 Ligand Preparation

The chemical composition of the essential oils was analyzed using GC–MS, following a previously established protocol by our research team. This analysis allowed the identification of major terpene compounds present in the oils obtained from *I. viscosa* grown under different climatic conditions (Laftouhi, Hmamou, et al. 2024), together with the reference inhibitors xanthene (CID: 7107) and acarbose (CID: 41774), which were retrieved from the PubChem database in 3D SDF format (available online at https://pubchem.ncbi.nlm.nih.gov/). The ligands intended for molecular docking against GR, α‐glucosidase, and α‐amylase were initially transformed into PDB format via PyMOL (version 2.5.3) to facilitate structural alignment (Farihi et al. 2023). Subsequently, the ligands were converted into PdbQ format using AutoDock Tools (ADT; version 1.5.7, The Scripps Research Institute) (Shaweta et al. 2021).

### 2.7 ADME Studies

Understanding pharmacokinetic parameters such as absorption, distribution, metabolism, and excretion (ADME) is essential to determine how a compound acts within the body. These parameters trace the journey of a substance from intake to elimination. Nowadays, computational approaches play a key role in predicting the ADME profiles of various molecules (Srivastava et al. 2020). They analyze membrane permeability, interaction with key transporters and enzymes, and metabolic stability. For our analysis, we chose the SwissADME platform (Available online: www.swissadme.ch) (Farihi et al. 2023). The platform allows for a detailed investigation of the physicochemical properties of *I. viscosa* essential oil phytocompounds, supports the evaluation of their therapeutic potential, and facilitates the prediction of pharmacokinetic parameters, providing a thorough overview of their ADME profiles.

### 2.8 Statistical Analysis

Data are shown as mean ± SEM. One‐way ANOVA followed by Tukey's test was used for statistical analysis, performed using GraphPad Prism 5 (San Diego, CA, USA). Distinct letters (a, b, ab, and c) were used in the respective figures and tables to indicate significant differences between the values. Different letters represent statistically significant differences, while shared letters indicate partial similarities. This approach provides a clear visual representation of the similarities and differences among the samples.

## 3 Results and Discussion

### 3.1 Antioxidant Activity of *Inula viscosa* Essential Oils

#### 3.1.1 DPPH Free Radical Scavenging Activity

**FIGURE 2:**
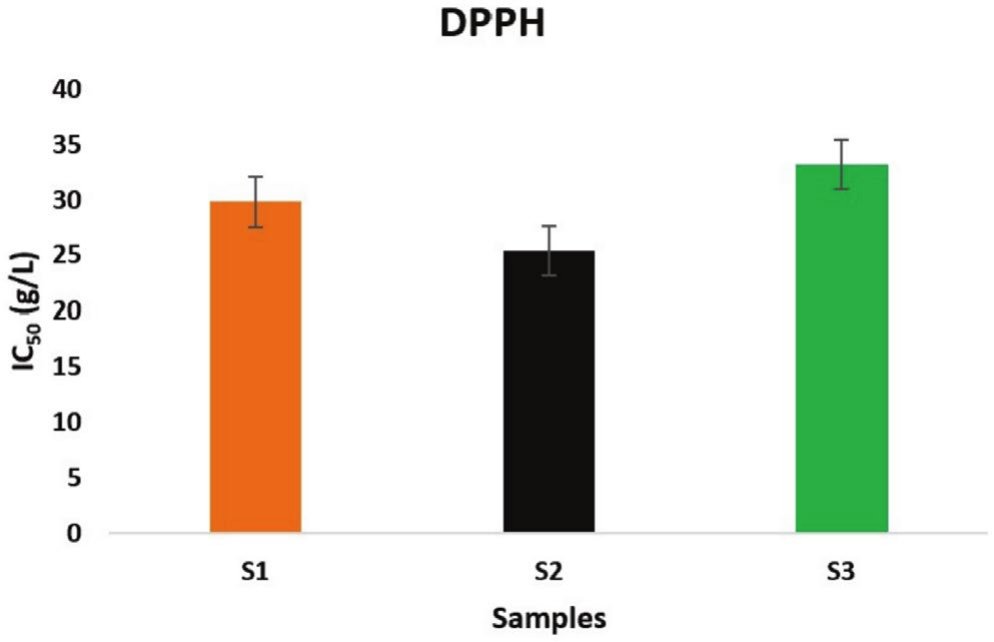
DPPH free radical scavenging activity IC_50_ (g/L) of *Inula viscosa* essential oils.

Figure 2 shows the antioxidant activity of *I. viscosa* essential oils under three different climatic conditions, as measured by the DPPH test. The results indicate that Sample 2 exhibits the strongest antioxidant activity, followed by Sample 1, and finally, Sample 3 (S1: IC_50_ = 29.8 ± 0.06 g/L, S2: IC_50_ = 25.4 ± 0.09 g/L, S3: IC_50_ = 33.2 ± 0.11 g/L). These findings demonstrate that changes in meteorological parameters, such as temperature and precipitation, can influence the biological activity of plants. Identically, changes in biological activities may be attributed to alterations in the chemical compositions of plants, which result from varying climatic parameters. These results are confirmed by Chrysargyris et al. (2020), who found that modifications in environmental conditions alter the content of essential oils, increase the levels of phenolic compounds, and enhance the antioxidant activity in *Artemisia* plants. Specific parameters also increased for other plants, such as total phenols in spearmint and flavonoids in rosemary. Consequently, Yeddes et al. (2018) and Liu et al. (2016) confirmed that the essential oil yield of rosemary samples from different climates is changed, and also there is a change in the chemical compositions of essential oils. The Pearson correlation matrix between the chemical classes of essential oils and the antioxidant capacity of the oil indicated that samples rich in oxygenated sesquiterpenes had significant antioxidant activity (Liu et al. 2023; Mssillou et al. 2022).

#### 3.1.2 ABTS Radical Scavenging Activity

**FIGURE 3:**
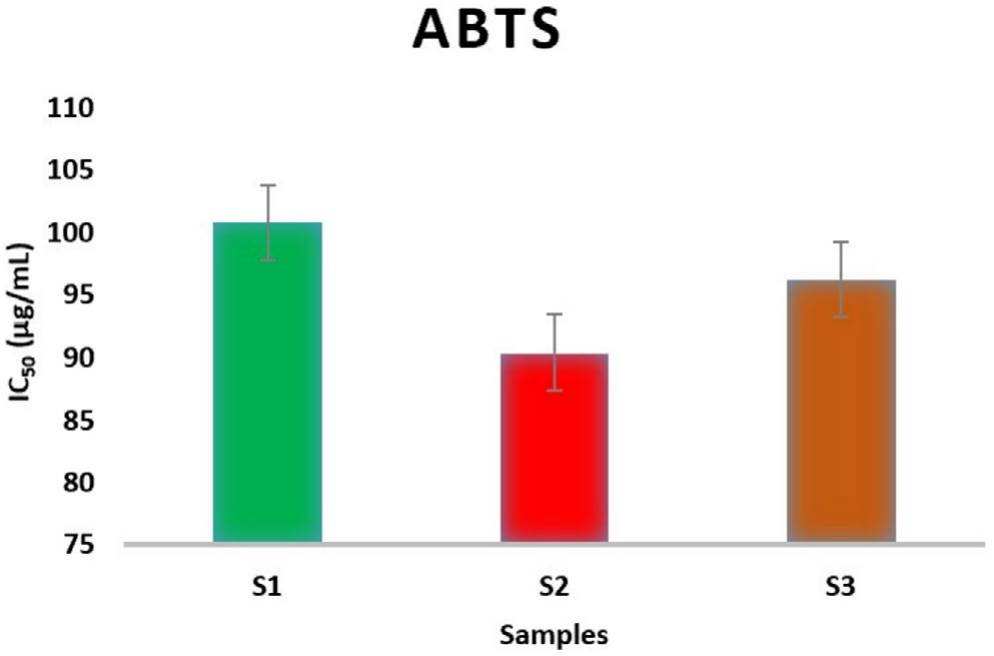
ABTS radical scavenging activity IC_50_ (μg/mL) of *Inula viscosa*.

Figure 3 represents the variation in the antioxidant activity of *I. viscosa* essential oils under three different climatic conditions, as measured by the ABTS test. The results in this figure show that the antioxidant activity of essential oils is influenced by variations in temperature and precipitation. As a result, Sample 2 exhibits the highest antioxidant activity, followed by Sample 3, with Sample 1 having the lowest (S1: IC_50_ = 100.8 ± 4.03, S2: IC_50_ = 90.4 ± 2.00, S3: IC_50_ = 96.2 ± 0.50 μg/mL). These three samples successively experience an increase in temperature and water stress levels. The modification of antioxidant activity may be due to a change in the chemical compositions of essential oils. Similarly, another study found that various environmental factors could affect the phytochemicals and antioxidant activity (Hafsé et al. 2015; Laftouhi, Eloutassi, Ech‐Chihbi, et al. 2023; Laftouhi, Slimani, et al. 2024). Temperature and altitude particularly influence the concentrations of certain compounds such as 1, 8‐cineole, limonene, and menthone, while average precipitation has a major impact on the levels of trans‐piperitone epoxide, piperitenone oxide, and pulegone. Our results demonstrate that these environmental factors play a significant role in determining the essential oil content and composition, as well as the antioxidant activity in *I. viscosa* (Asraoui, Kounnoun, Cadi, et al. 2021).

#### 3.1.3 Essential Oil β‐Carotene Bleaching Test

**FIGURE 4:**
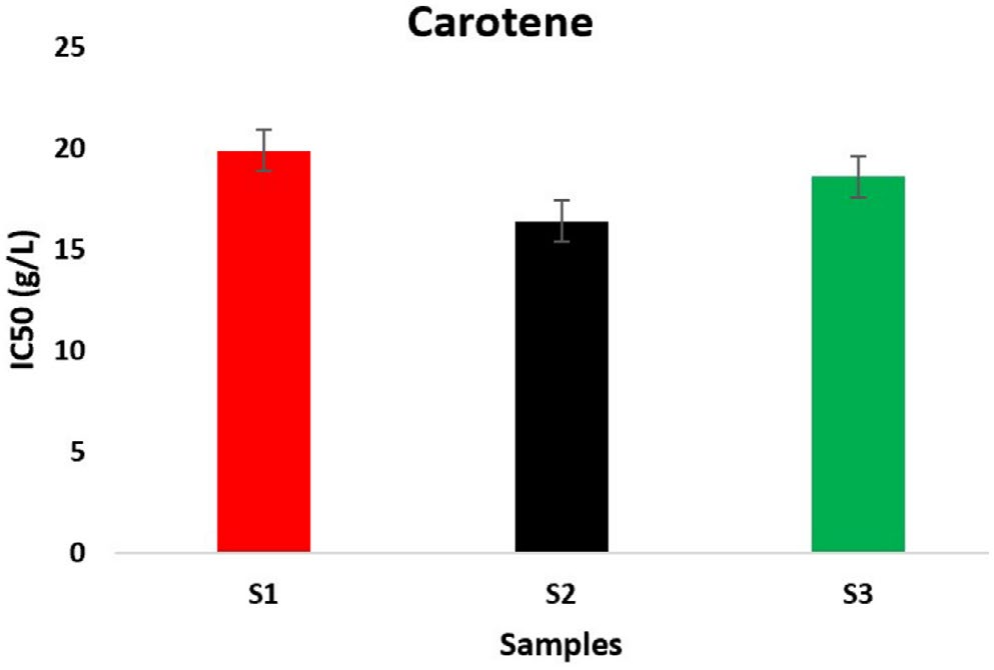
Carotene bleaching test IC_50_ (g/L) of *Inula viscosa* essential oil.

We note from Figure 4, which represents the variation in the antioxidant activity of three samples of *I. viscosa* under three climatic conditions measured by the carotene bleaching test, that Sample 2 has strong antioxidant activity. This sample experienced a 5°C increase in temperature and 50% water stress. It is followed by Sample 3, which experienced a 10°C increase in temperature and 75% water stress. Finally, sample 1, planted under normal temperature and precipitation conditions, exhibits the lowest antioxidant activity (S1: IC_50_ = 19.9 ± 0.10, S2: IC_50_ = 16.4 ± 0.06, S3: IC_50_ = 18.6 ± 0.15 g/L). Previous studies have also discovered that environmental stresses and the origin of plants affect the yield of essential oils (Gharred et al. 2022), their chemical compositions, and their antioxidant activity, supporting the results found in this study (Laftouhi, Eloutassi, Drioua, et al. 2023; El‐Hilaly et al. 2003; Laftouhi, Eloutassi, Ech‐Chihbi, et al. 2023).

### 3.2 Antidiabetic Activity of *Inula viscosa* Essential Oil

Diabetes is a chronic autoimmune disease that affects approximately 8.5% of the population; it affects adults and children. Diabetes can lead to either hypoglycemia or hyperglycemia (Mekala and Bertoni 2020). In healthy individuals, two hormones regulate blood glucose levels: insulin, which lowers blood glucose concentration, and glucagon, also known as the fasting hormone, which raises blood glucose concentration. Diabetes encompasses several types, with the most prevalent being type 1 and type 2. Type 1 diabetes, also known as insulin‐dependent or juvenile diabetes, arises from inadequate insulin production due to the autoimmune destruction of beta cells in the pancreas. In contrast, type 2 diabetes, often linked to insulin resistance, occurs when cells in the body become less responsive to insulin (Flores Monar et al. 2022). These conditions lead to dysregulation of blood glucose levels, contributing to various health complications if not managed effectively (Sanyaolu et al. 2023). Currently, rather than relying solely on pharmaceutical drugs, many people are opting for treatment using aromatic and medicinal plant products. Therefore, the analysis of the results presented in Table 1, which illustrates the variation in the antidiabetic activity of three samples of *I. viscosa* plant under different climatic conditions, uses two tests: α‐amylase inhibitory activity and α‐glucosidase inhibitory activity. Therefore, regarding α‐amylase inhibitory activity, S2 exhibits the strongest antidiabetic activity, followed by S3 and then S1 (S1: IC_50_ = 47.76 ± 0.05 μg/mL, S2: IC_50_ = 40.3 ± 0.07 μg/mL, S3: IC_50_ = 43.1 ± 0.1 μg/mL). Similarly, for α‐glucosidase inhibitory activity, the priority order is S2, S3, and S1 (S1: IC_50_ = 24209 ± 3.45 μg/mL, S2: IC_50_ = 20106 ± 2.66 μg/mL, S3: IC_50_ = 22012 ± 4.66 μg/mL). These variations in *I. viscosa's* antidiabetic activity across the two tests are primarily attributed to changes in the plant's metabolic profile and the chemical compositions of its essential oils. Similarly, Asraoui et al. (2021) discovered that *I. viscosa* leaves exhibit a significant inhibitory effect on α‐amylase and α‐glucosidase. Additionally, previous studies (Eruygur et al. 2024; Yildirim et al. 2022) demonstrate that recent in vitro research on *I. viscosa* has shown strong antioxidant activity, along with notable inhibitory effects on α‐glucosidase and α‐amylase.

**TABLE 1:** Antidiabetic activity of *Inula viscosa* essential oil.

	α‐Amylase inhibitory activity IC_50_ (μg/mL)			α‐Glucosidase inhibitory activity IC_50_ (μg/mL)		
Samples	S1	S2	S3	S1	S2	S3
	47.76 ± 0.05^a^	40.3 ± 0.07^b^	43.1 ± 0.1^ab^	242.09 ± 3.45^a^	201.06 ± 2.66^b^	220.12 ± 4.66^ab^
Acarbose	4.85 ± 0.11^c^			40.06 ± 2.21^c^		

*Note:* Different letters (a, b, c) indicate values that are significantly different from each other (*p* < 0.05). Shared letters (e.g., “ab”) denote partial similarities between values.

### 3.3 Molecular Docking Results

#### 3.3.1 Antidiabetic Activity: Interactions With Human Enzymes α‐Amylase (PDB ID: 1B2Y) and α‐Glucosidase (PDB:5NN8)

**FIGURE 5:**
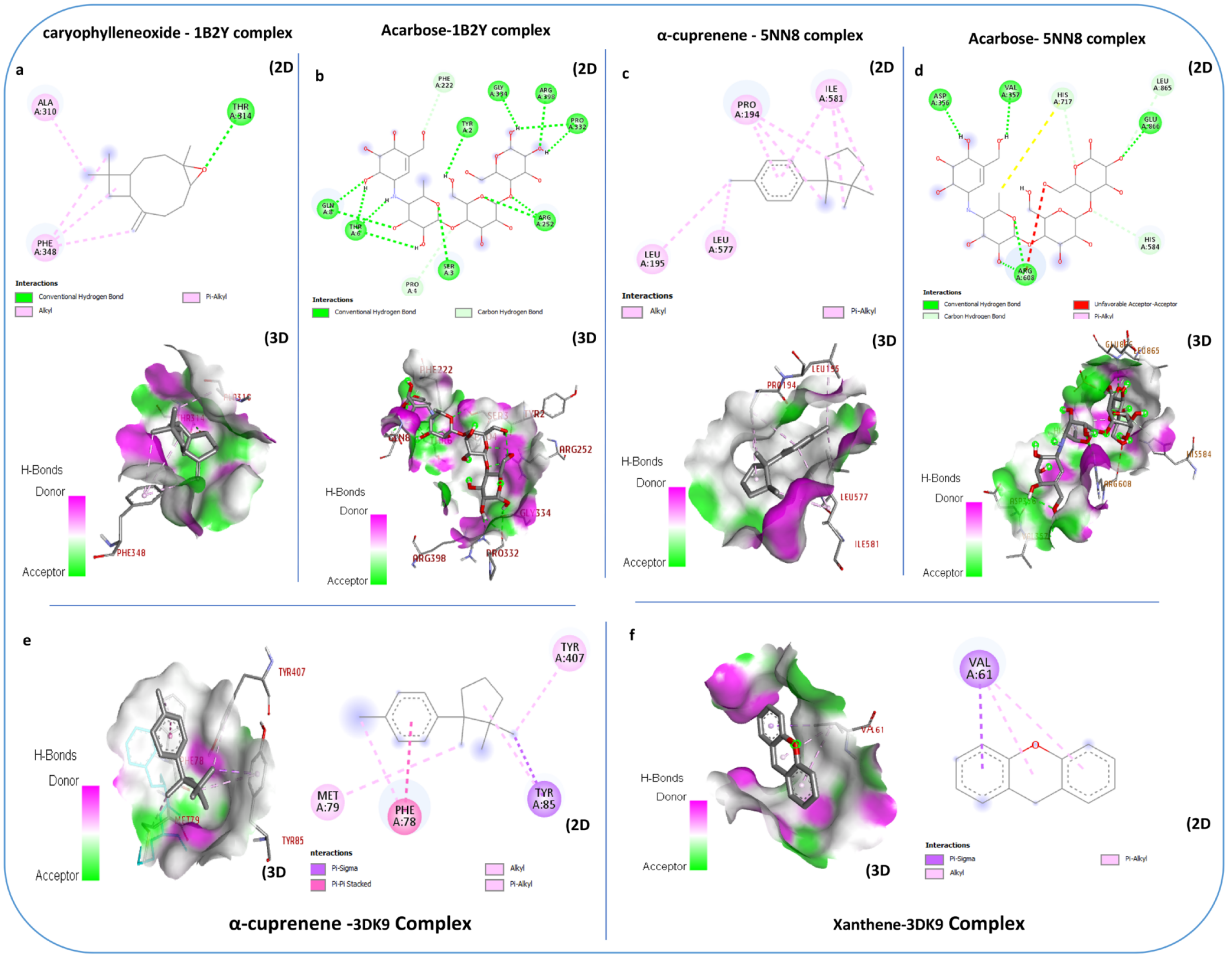
2D and 3D binding interactions of caryophyllene oxide and α‐cuprenene with human α‐amylase (PDB: 1B2Y) (a), human α‐glucosidase (PDB: 5NN8) (c), and glutathione reductase (PDB: 3DK9) (e) were compared to the standard inhibitors acarbose and xanthine (b, d, f).

α‐Amylase is a crucial enzyme in carbohydrate metabolism, significantly contributing to the digestion process by cleaving 1,4‐glycosidic bonds in starch. Numerous studies have explored the enzyme as a therapeutic target for diabetes treatment (Sahnoun et al. 2017). As well as, the importance of α‐amylase extends beyond digestion, highlighting its potential as a target for improving diabetes management and emphasizing its relevance in medical and therapeutic settings (Li et al. 2018). In this study, the results obtained from the molecular docking of ligands to α‐amylase (PDB: 1B2Y) revealed that all phytochemical compounds showed weak or similar affinities for α‐amylase. However, caryophyllene oxide demonstrated an affinity closer to that of the antidiabetic agent acarbose (−8.2 kcal/mol), (Table 2) shows that the compound formed stable interactions with several amino acid residues of the enzyme. Notably, it established hydrogen bonds with Thr314 (Figure 5a). These findings suggest that caryophyllene oxide holds significant promise as a potential antidiabetic agent warranting further investigation. α‐Glucosidase is a crucial enzyme in carbohydrate metabolism, responsible for catalyzing the hydrolysis of α‐glucose residues from the non‐reducing ends of polysaccharides such as starch and disaccharides. This enzymatic action converts complex carbohydrates into monosaccharides, facilitating their absorption into the bloodstream and directly influencing glycemic levels. In addition to its physiological role in digestion, α‐glucosidase serves as a valuable pharmacological target in diabetes therapy, underlining its dual importance. In this study, molecular docking analyses with α‐glucosidase (PDB ID: 5NN8) demonstrated that the tested phytochemicals exhibited either weak or comparable binding affinities when assessed against α‐amylase. However, α‐cuprenene demonstrated greater affinity than the antidiabetic agent acarbose (−7.2 kcal/mol) (Table 2) and interacted effectively with several amino acid residues of the enzyme. Specifically, it exhibited pi‐alkyl and alkyl bonding interactions with Pro 194, Leu 195, Leu 577, and Ile 581. These results highlight the promising potential of caryophyllene oxide as a candidate deserving further exploration as an antidiabetic agent. The docking results and in vitro antidiabetic activity of *I. viscosa* essential oils are closely interconnected, providing a comprehensive understanding of the potential antidiabetic properties of its phytochemical constituents. The molecular docking studies revealed that certain compounds, such as caryophyllene oxide and α‐cuprenene, exhibit promising affinities for α‐amylase and α‐glucosidase, with caryophyllene oxide showing an affinity comparable to acarbose for α‐amylase and α‐cuprenene demonstrating greater affinity than acarbose for α‐glucosidase. These findings are supported by the in vitro assays, which showed that *I. viscosa* essential oils possess significant α‐amylase and α‐glucosidase inhibitory activities, with sample S2 exhibiting the strongest antidiabetic effects in both tests. The correlation between the docking affinities and the in vitro inhibitory activities highlights the potential of these compounds as effective antidiabetic agents and underscores the value of combining computational and experimental approaches to evaluate their therapeutic potential.

**TABLE 2:** Molecular binding affinities (kcal/mol) between phytochemicals identified in essential oils of *Inula viscosa* with α‐amylase (PDB: 1B2Y), α‐glucosidase (PDB: 5NN8) and glutathione reductase (PDB: 3DK9).

	Antidiabetic		Antioxidant
	α‐Amylase (1B2Y)	α‐Glucosidase (5NN8)	GR (3DK9)
	Free binding energy (kcal/mol)		
Inhibitor standard	−8.2	−7.2	−6.2
α‐Cedrene	−6.5	−5.8	−7.9
(E)‐Caryophyllene	−6.1	−5.9	−8.3
δ‐Selienene	−6.2	−6.5	−8.2
α‐Cuprenene	−5.7	7.3	−9.2
α‐Calacorene	−6.0	−6.3	−8.6
(E)‐Nerolidol	−4.8	−5.6	−6.8
Caryophylleneoxide	−7.7	−6.1	−7.9
1‐Hexadecene	−4.0	−4.5	−5.7
Guaiol	−6.1	−6.0	−8.5

#### 3.3.2 Antioxidant Activity: Interactions With Human Enzyme Glutathione Reductase (PDB: 3DK9)

Glutathione reductase (GR) is an essential enzyme that plays a critical role in protecting cells from oxidative stress. Inhibiting GR and the regeneration of glutathione (GSH), a major antioxidant, may provide therapeutic benefits by enhancing excessive oxidative stress, which is associated with cells causing various diseases. This approach takes advantage of the reliance of cancer cells and some microorganisms on their pathological GR enzymes for their antioxidant defenses. When applied judiciously, this strategy can enhance the effectiveness of other treatments, leading to a more focused and potent method for addressing cancer and microbial infections. In this study, the results obtained from the molecular docking of ligands to the healthy human enzyme glutathione reductase (PDB: 3DK9) revealed that all molecules displayed greater affinity than xanthene (−6.2 kcal/mol) (Table 3), except for 1‐hexadecene, which showed a weaker affinity than the standard inhibitor. The molecule α‐cuprenene stood out due to its strong affinity for glutathione reductase, interacting effectively with several amino acid residues of the enzyme. Specifically, it demonstrated Pi‐sigma, Pi‐alkyl, alkyl, and Pi‐Pi stacked bonding interactions with Phe 78, Met 79, Tyr 85, and Tyr 407. This higher affinity is significant because it is often correlated with increased inhibitory activity. These results highlight the promising potential of α‐cuprenene as a candidate deserving further exploration as an inhibitor of pathological GR enzymes from cancer and microbial cells, without significant inhibition of the healthy human GR enzyme, under appropriate assay conditions in vitro and in vivo (Ferreira et al. 2024; Korkmaz 2024).

**TABLE 3:** *In silico* analysis of the pharmacokinetic properties (ADME) of phytochemicals identified in essential oils of *Inula viscosa* .

	Physicochemical properties					Lipophilicity		Druglikeness		Pharmacokinetics		
Compounds	MW g/mol	HBA	HBD	TPSA Å^2^	Rotatable Bonds	M logP	W logP	Lipinski's	Verbier's	BBB Permeation	GI absorption	CYP1A2 inhibitor
α‐Cedrene	204.3	0	0	0.0	0	5.6	4.4	1	0	No	Low	No
(E)‐Caryophyllene	204.3	0	0	0.0	0	4.6	4.7	1	0	No	Low	No
δ‐Selienene	204.3	0	0	0.0	1	4.6	4.8	1	0	No	Low	No
α‐Cuprenene	204.3	0	0	0.0	1	4.6	4.8	1	0	No	Low	No
α‐Calacorene	200.3	0	0	0.0	1	5.3	4.5	1	0	No	Low	No
(E)‐Nerolidol	222.3	1	1	20.2	7	3.8	4.4	0	0	Yes	High	Yes
Caryophylleneoxide	220.3	1	0	12.5	0	3.6	3.9	0	0	Yes	High	No
1‐Hexadecene	224.4	0	0	0.0	13	6.2	6.2	1	1	No	Low	Yes
Guaiol	222.3	1	1	20.2	1	3.6	3.9	0	0	Yes	High	No

### 3.4 ADME Analysis Results

**FIGURE 6:**
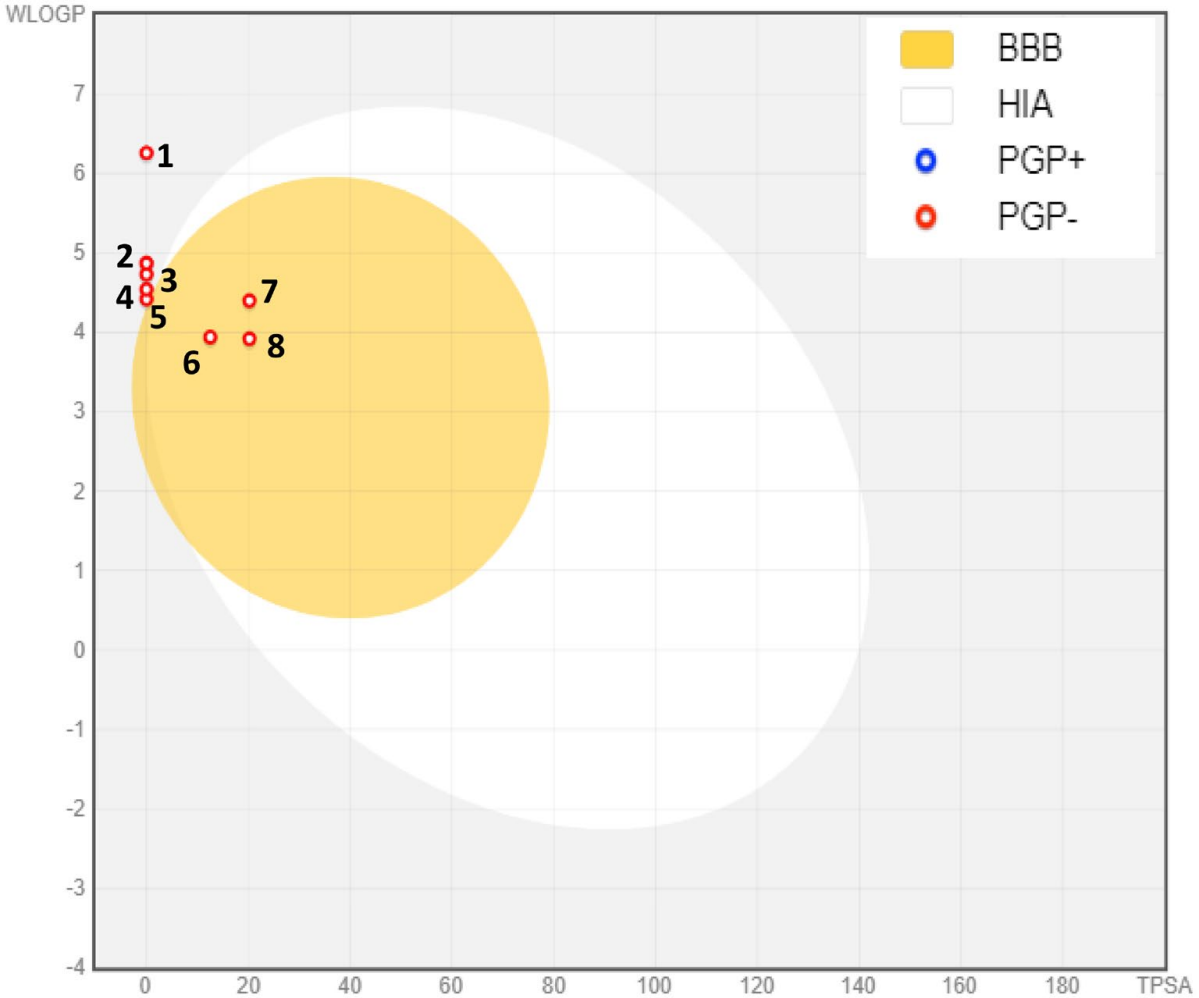
Boiled‐egg model of the blood–brain barrier (BBB) permeability and gastrointestinal absorption of phytochemicals identified in essential oils of *Inula viscosa*. (1) 1‐Hexadecene, (2) δ‐selienene, (3) (*E*)‐caryophyllene, (4) α‐calacorene, (5) α‐cedrene, (6) caryophylleneoxide, (7) (*E*)‐nerolidol, (8) guaiol. PGP‐: Non‐substrate of P‐glycoprotein, PGP+: P‐glycoprotein substrate.

**FIGURE 7:**
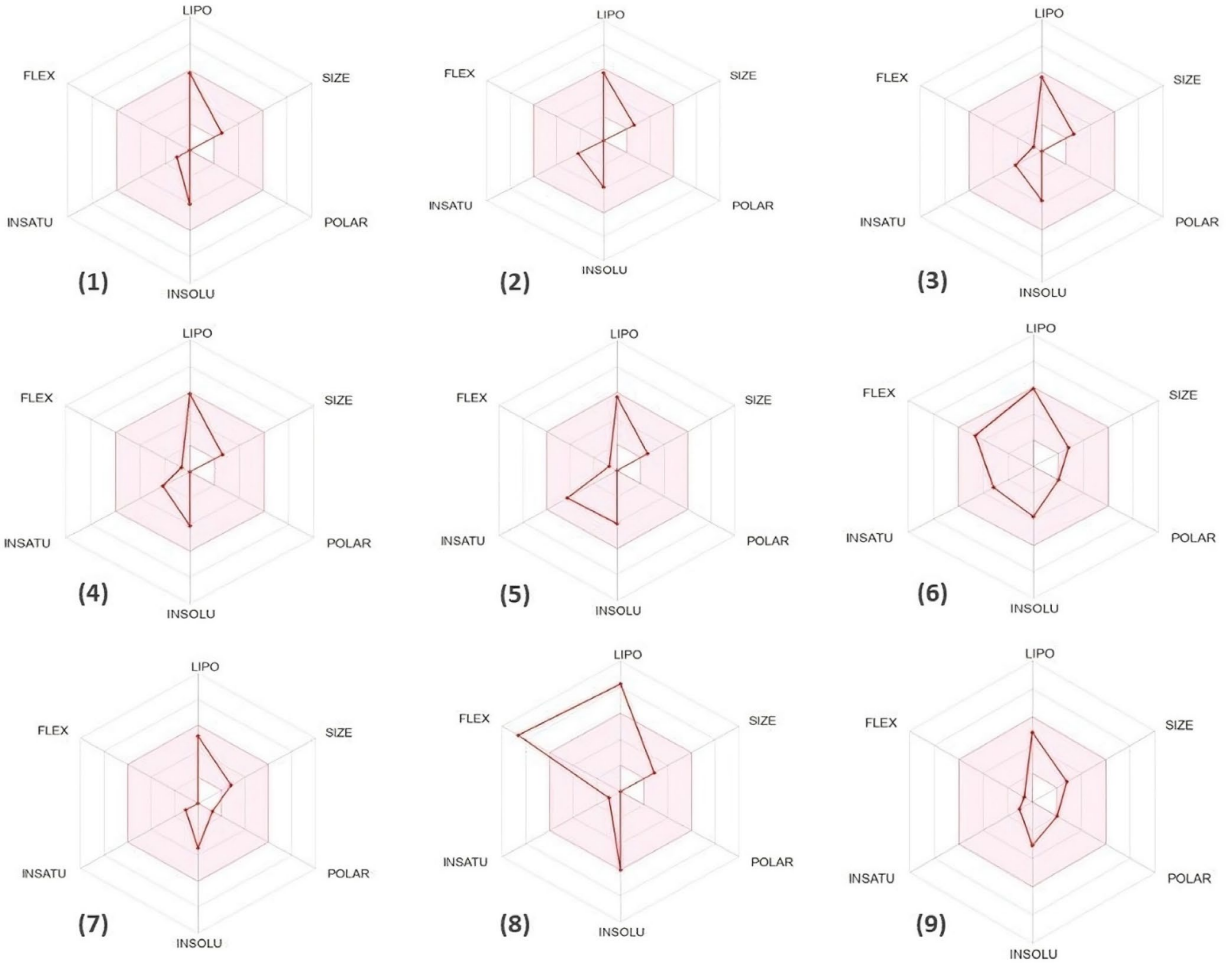
Bioavailability radar of phytochemicals identified in essential oils of *Inula viscosa* . (1) α‐Cedrene, (2) (E)‐caryophyllene, (3) δ‐selienene, (4) α‐cuprenene, (5) α‐calacorene, (6) (E)‐nerolidol, (7) caryophyllene oxide, (8) 1‐hexadecene, (9) guaiol.

Preliminary pharmacokinetic evaluations through computational modeling facilitate the rapid identification of promising drug candidates and streamline the drug development process. Such analyses contribute to minimizing adverse effects, decreasing the chances of failure in drug development, and improving success rates in clinical trials (Farihi et al. 2023). To be considered suitable for oral drug development, compounds are generally expected to comply with Lipinski's rule of five and Veber's rule, allowing at most one violation among the following criteria: (1) no more than 10 hydrogen bond acceptors (oxygen or nitrogen atoms), (2) an octanol–water partition coefficient (MLogP) below 5, (3) a molecular weight under 500 Da, and (4) no more than 5 hydrogen bond donors (Lipinski et al. 2012). In our study, we observed that all phytochemicals identified in essential oils of *I. viscosa* examined met Lipinski's criteria, indicating their potential suitability for oral drug development. 1‐Hexadecene exhibits a violation of Veber's rule, as it has several rotatable bonds greater than 10. Our investigation showed that (E)‐nerolidol (TPSA = 20.2 Å^2^, WLogP = 4.4), caryophyllene oxide (TPSA = 12.5 Å^2^, WLogP = 3.9), and guaiol (TPSA = 20.2 Å^2^, WLogP = 3.9) can cross the blood–brain barrier (BBB) (Table 3). This ability to penetrate is due to its low polarity and moderate lipophilicity, enabling efficient distribution throughout brain tissue (Figure 6) (Daina and Zoete 2016). Furthermore, all compounds analyzed have been identified as not being substrates of P‐glycoprotein (PGP‐) (Figure 6); this implies that they may escape early elimination by PGP, allowing them to maintain more stable and prolonged therapeutic concentrations within the bloodstream (Daina et al. 2017). A molecule with high intestinal absorption provides considerable benefits in terms of bioavailability, efficacy, convenience, and tolerance, making it a strong candidate for oral drug development (Stillhart et al. 2020). Specifically, (E)‐nerolidol, caryophyllene oxide, and guaiol exhibited high intestinal absorption (Table 3). Cytochrome P450, a crucial enzyme for detoxification, is primarily located in the liver (Tascioglu Aliyev et al. 2021). Our analysis showed that all of the compounds examined are neither inhibitors nor substrates of CYP450 enzymes, specifically CYP1A2, except for (E)‐nerolidol and 1‐hexadecene (Table 3). This indicates a reduced likelihood of these phytochemicals interfering with drug metabolism, thereby improving their safety profiles. Figure 7 presents bioavailability radars for the identified compounds, offering a visual summary of their oral bioavailability potential. The pink area on the radar defines the optimal physicochemical space in which the molecule's profile must fit entirely to be considered drug‐like. This criterion is essential for assessing a compound's therapeutic viability, as it reflects its potential for effective absorption and systemic distribution (Daina et al. 2017). Notably, the bioavailability radar for all the examined compounds falls within the pink zone, except for 1‐hexadecene, indicating their potential as drug candidates.

The study of the impact of climate change on the chemical compositions and biological activities of plants presents several significant limitations. The natural variability of plants, influenced by factors such as season, soil, altitude, and agricultural practices, makes it challenging to attribute observed changes exclusively to climate change. Moreover, short‐term studies may not fully capture long‐term effects, and experiments conducted in controlled environments, such as laboratories or greenhouses, may not accurately reflect natural conditions. Additionally, changes in chemical compositions do not necessarily translate directly into variations in biological activities, as other biological or environmental factors may intervene. In this context, it is crucial to emphasize the importance of combining computational and experimental studies. In silico approaches, such as molecular modeling and simulations, allow for the prediction of interactions, mechanisms of action, and compound bioavailability, thus reducing research time and costs. Complementarily, in vitro studies validate these predictions under controlled biological conditions, ensuring the reliability of the results. The integration of these two methodologies provides a synergistic approach that yields more robust conclusions and helps to overcome some of the inherent limitations of this type of research (Bédoui et al. 2024; Rahmouni et al. 2024).

## 4 Conclusion

This study investigated the intricate interplay between climatic conditions, chemical composition, and the antioxidant and antidiabetic activities of essential oils from *I. viscosa* samples grown under different climatic conditions. The chemical composition of the essential oils exhibited considerable variability, with the proportions of key compounds being influenced by the climatic cultivation conditions. This highlights the significant impact of environmental factors on the biochemical composition of essential oils. The essential oils of *I. viscosa* demonstrated significant antioxidant activity, as evidenced by their ability to neutralize DPPH and ABTS radicals. The essential oil samples also showed varying levels of antidiabetic activity, attributed to their differential abilities to inhibit the enzymes α‐amylase and α‐glucosidase. The antioxidant and antidiabetic activities of these oils can be primarily attributed to their major bioactive compounds, which are recognized for their beneficial properties. The application of ANOVA and Tukey's tests confirmed significant differences in antioxidant and antidiabetic activities among the essential oil samples, with the samples from the arid climate showing the most potent effects. In silico analysis further supported these findings by revealing that the compounds exhibited varying affinities for the target enzymes, underscoring their potential as effective antioxidants and antidiabetics. Additionally, ADME analysis indicated that several of these compounds possess favorable pharmacokinetic properties for oral administration. Furthermore, in the context of glutathione reductase (GR), the study emphasizes the importance of selective inhibition, where compounds were shown to interact preferentially with pathological GR enzymes over healthy human GR, suggesting potential therapeutic applications in conditions like cancer and microbial infections. This study enhances our understanding of how climatic conditions influence the chemical composition and biological activity of *I. viscosa* essential oils. The findings suggest that arid climates generally favor the production of chemical compounds with superior antioxidant and antidiabetic activities, offering promising directions for future research and applications.

## Author Contributions

Conceptualization, A.L., and A.E., methodology, A.L., A.F.; A.E., M.B., software, A.F., N.E., and A.H.; validation, F.A.N., M.B., M.A., and A.A.Q.; formal analysis, M.A.M., N.E., and A.T.; investigation, A.T., and M.T.; resources, F.A.N., A.T., and M.T.; data curation, A.H. and A.T.; writing – original draft preparation, A.L., A.F., and A.E.; writing – review and editing, A.E., M.A.M., M.B., F.A.N., A.F., M.A., A.A.Q., N.E., A.T., A.H., and M.T.; visualization, M.A.M., M.A., and A.A.Q.; supervision, M.T.; funding acquisition, F.A.N., M.A., and A.A.Q.; project administration, M.T. All authors have read and agreed to the published version of the manuscript.

## Conflicts of Interest

The authors declare no conflicts of interest.

## Data Availability

Data supporting the findings of this study can be obtained from the corresponding author, Abdelouahid Laftouhi, upon reasonable request.
